# Application of sage and ginger extracts in dry fermented salami

**DOI:** 10.1016/j.fochx.2025.102809

**Published:** 2025-07-17

**Authors:** Filip Hruška, Filip Beňo, Gabriela Krátká, Rudolf Ševčík

**Affiliations:** University of Chemistry and Technology, Prague, Faculty of Food and Biochemical Technology, Department of Food Preservation, Technická 5, 166 28 Prague 6 – Dejvice, Czech Republic

**Keywords:** Antioxidants, Fermented meat products, Ginger, Plant extracts, Sage

## Abstract

Meat products are prone to lipid oxidation, which affects their quality. Although synthetic antioxidants are effective, plant extracts rich in phenolic compounds offer a promising natural alternative. This study evaluated ethanol extracts and essential oils from sage and ginger for their antioxidant potential. Ethanol extracts with the highest phenolic content—60 % sage and 80 % ginger—were selected alongside the most active essential oils. These were applied to dry fermented sausages to assess antioxidant effects. Ethanol extracts at a 2 % concentration significantly reduced lipid oxidation, with up to 75 % reduction in malondialdehyde levels compared to the control. Essential oils exhibited the strongest in vitro antioxidant activity, while ethanol extracts demonstrated better phenolic content. Substantial moisture loss during storage, indicated by water activity, likely influenced the results and highlighted the importance of controlled ripening. Overall, sage and ginger extracts showed potential as natural antioxidants in meat systems.

## Highlights

• Ethanol extracts had the highest phenolic content (60 % sage, 80 % ginger).
• Fresh essential oils of sage and ginger showed the strongest antioxidant activity.
• DPPH results highlight the need for methodological standardization.
• Plant extracts reduced oxidation in fermented meat at higher inclusion levels.

## Graphical abstract

**Figure:**
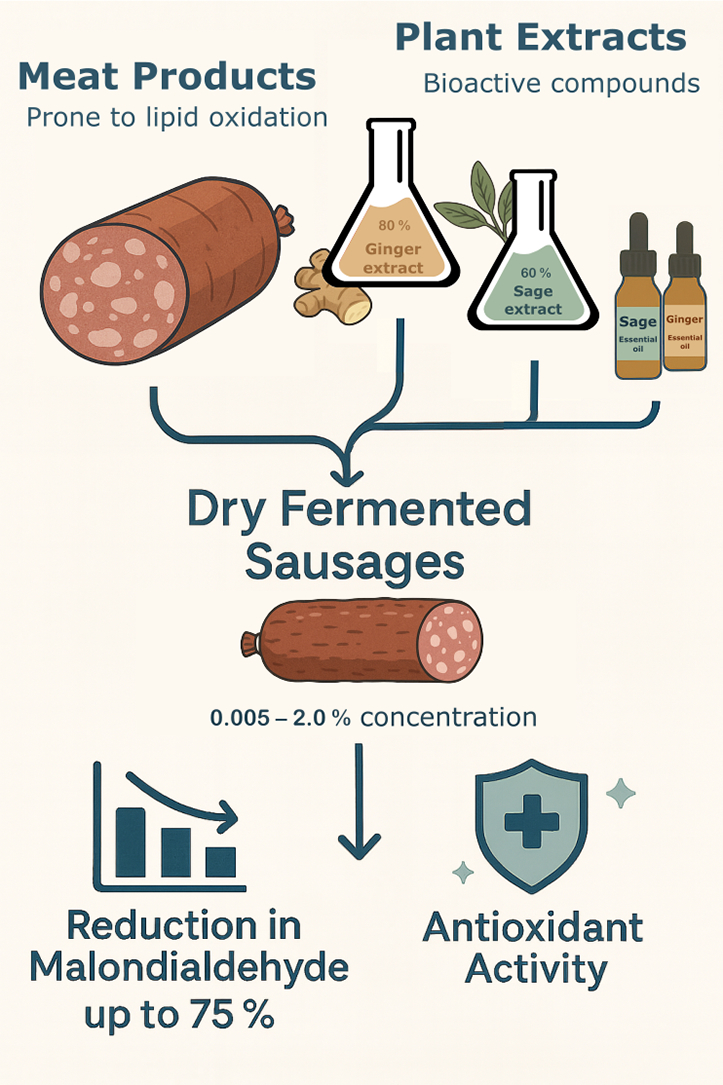

## 1 Introduction

The shelf-life of meat and meat products is primarily influenced by lipid oxidation and microbial spoilage (Cao et al., 2013). Dry fermented salami, characterized by their high fat content and extended ripening periods, are particularly susceptible to lipid oxidation, which can lead to rancidity and undesirable sensory changes (Šojić et al., 2014). Additionally, the absence of thermal treatment during dry fermented salami production increases the risk of microbiological contamination, including foodborne pathogens such as *Listeria monocytogenes* (Mohamed et al., 2022).

To limit these issues, antioxidants and preservatives are routinely added to meat products (Franco et al., 2019). In the European Union, such additives must be declared on the label using *E*-numbers (European Parliament and Council of the European Union, 2011). Although these substances have been scientifically evaluated and deemed safe at permitted levels, they are often perceived negatively by consumers, who associate them with artificial or harmful effects (Bearth et al., 2014). However, according to experts, the levels used in food products are well below safety thresholds and do not pose any health risk (Franco et al., 2019).

Plant extracts and essential oils are increasingly explored for their antioxidant and antimicrobial properties, and are generally perceived as safer and more natural by consumers (Aminzare et al., 2019; Carocho et al., 2015; Munekata et al., 2020). In addition to their shelf-life extending potential, some plant compounds have been shown to offer health benefits and may contribute to reducing the need for synthetic nitrites (Ferysiuk & Wójciak, 2020). Recent trends in consumer behavior show a growing preference for food products with natural ingredients, especially those perceived to improve health and reduce chemical additives, which further increases the demand for plant-based preservatives (Kumar et al., 2015).

Sage (*Salvia officinalis* L.) and ginger (*Zingiber officinale*) are medicinal plants known for their strong antioxidant and antimicrobial activities. Sage contains compounds with antioxidant activity such as carnosol, carnosic acid, limonene, geraniol, α-pinene, borneol, and eucalyptol (Yazgan, 2020; D. Zhang et al., 2022). The concentration of these compounds varies depending on geographical origin, harvest time, and extraction conditions, which in turn affect their biological activity, including antioxidant and antimicrobial properties (Olszewska et al., 2020; Ong, 2004). Several of these compounds, including α-pinene and eugenol, have shown confirmed antimicrobial effects (Borges et al., 2022; Hoch et al., 2023). Ginger contains active constituents such as gingerol, shogaol, zingerone, zingiberene, and linalool, many of which contribute to its bioactivity and sensory properties (Beristain-Bauza et al., 2019; D. Zhang et al., 2022). Zingerone has been linked to antibacterial effects (Larijanian et al., 2024), while 6-shogaol has demonstrated antiviral, anti-biofilm, and antimicrobial activities (Lee et al., 2018; Mao et al., 2019).

The inclusion of plant extracts in meat products typically ranges from 0.05 % to 2 % of the total product weight and primarily depends on the extract's antioxidant capacity, the desired increase in oxidative shelf life, and the sensory compatibility with the meat product (Munekata et al., 2020). At higher concentrations, sensory characteristics such as color, aroma, and taste may be affected during storage (Nikmaram et al., 2018).

These natural extracts are increasingly studied as potential alternatives to synthetic additives in food preservation, particularly in meat products, due to their multifunctional bioactivities.

Therefore, the aim of this study was to evaluate the potential of produced sage and ginger extracts—specifically essential oils and ethanol-based extracts—to enhance oxidative stability in dry fermented salami during ripening and storage. The study first focused on optimizing extraction parameters for both plants, followed by selection of the most promising extracts based on their antioxidant activity. These optimized extracts were subsequently incorporated into model DFS. Their effects on oxidative stability, water activity, and color development were systematically monitored over a five-week storage period.

## 2 Materials and methods

### 2.1 Reagents, materials, and equipment

The following chemicals were used in this study: ethanol (96 %, fermentation grade, p.a.), methanol (> 99.8 %), and acetic acid (99 %, p.a.) were obtained from Penta (Prague, Czech Republic); sodium carbonate (99 %, p.a.) and hydrochloric acid (35 %, p.a.) were purchased from Lach-Ner (Neratovice, Czech Republic); gallic acid monohydrate (≥ 98.0 %), 2,2-diphenyl-1-picrylhydrazyl, and l-ascorbic acid were supplied by Sigma-Aldrich (St. Louis, MO, USA); Folin–Ciocalteu phenol reagent was obtained from Supelco (Bellefonte, PA, USA); and 2-thiobarbituric acid (> 98.0 %) was purchased from Fluka (Buchs, Switzerland).

The analytical equipment used included an A&D HF-400 analytical balance (A&D Company, Limited, Japan), AQUALAB 4TEV water activity meter (Meter Group, Inc., USA), orbital shaker Varioshake VS 8 O (Lauda, Germany), Genesys 180 spectrophotometer (Thermo Scientific, USA), meat grinder HL - G 12 SS (distributor MASO-PROFIT Ltd., Czech Republic), BÜCHI K-355 distillation unit (SpectraLab Scientific Inc., Canada), CM-5 spectrophotometer (Konica Minolta, Japan), Liebherr GKPv 1470 refrigerator (Liebherr, Switzerland), thermometer and hygrometer TESTO 608-H1 (Testo SE & Co. KGaA, Germany).

Dried cut sage leaves (supplier: From Nature; harvested: Albania, 2023) were obtained from a local online vendor. Although the material was labeled as *Salvia officinalis*, no analytical verification of botanical purity was performed. Commercial samples of essential oils (ginger and sage, Phytos) were purchased from the same supplier and used solely for preliminary screening of antioxidant activity. Fresh ginger roots were purchased from a local supermarket. Beef and pork used in the preparation of the dry fermented salami were sourced from a local meat processing facility to ensure consistent quality. Additional ingredients required for salami production were obtained from local online suppliers. The detailed composition and formulation of dry fermented salami are provided in Table 1.

**Table 1:** Formulation of the model fermented meat product (lovecký salám).

Ingredients	m (kg)
Beef topside	4.03
Pork loin	3.44
Boneless pork shoulder	1.34
Boneless, skinless pork belly	7.34
Nitrite curing mix	0.344
Crushed black pepper	0.035
Sugar	0.018
Dried garlic	0.008
Starter culture	0.0025

### 2.2 Preparation of plant extracts and essential oils

Ethanol extracts were prepared following the methodology described by Durling et al. (2007), Mustafa and Chin (2023). The ginger root was washed, sliced (< 5 mm), and dried in a fruit dryer at 60 °C for 3.5 h. The slices were then ground using a spice mill. The water activity of the resulting plant materials was measured as 0.503 ± 0.009 for sage and 0.692 ± 0.014 for ginger. Three different ethanol concentrations (60 %, 70 %, and 80 %, *v*/v) were used for extraction. The dried material was extracted in 25 mL of ethanol solution (1,10 ratio) at laboratory temperature for 24 h using a shaker (300 rpm). The extracts were then evaporated to dryness and reconstituted in 80 % ethanol to a standardized concentration of 20 mg/mL (ginger) and 50 mg/mL (sage). The samples were stored at 4 °C until further analysis.

Essential oils were obtained by steam distillation using a Clevenger-type apparatus, following the procedures described by Mattje et al. (2019) for ginger and Zheljazkov et al. (2014) for sage. Dried ginger (10 g) was placed in a boiling flask with 400 mL of distilled water, and distillation was carried out for 3 h, yielding approximately 1 % oil. Dried sage (100 g) was distilled for 40 min, yielding approximately 0.8 % oil. The essential oil samples were stored at 4 °C until further analysis.

### 2.3 Determination of total phenolic content of plant extracts

Total phenolic content was determined according to the validated method of Kupina et al. (2018). A total of 7.5 mL of distilled water, 0.5 mL of the sample (for essential oils, a 500-fold diluted methanolic solution was used), and 0.5 mL of Folin-Ciocalteu phenol reagent were added to a test tube. After six minutes, 1.5 mL of sodium carbonate solution (20 %, *w*/*v*) was added, and the tube was shaken. The reaction was allowed to proceed for 2 h at 30 °C in a water bath. The absorbance of the solution at 765 nm was determined spectrophotometrically. The calibration curve was prepared using the same procedure with different concentrations of gallic acid. The results were expressed as gallic acid equivalents per dry weight (mg GAE/1 g DW). Each determination was performed in four replicates.

### 2.4 Determination of antioxidant activity (DPPH assay)

The determination of DPPH (2,2-Diphenyl-1-picrylhydrazyl) radical-scavenging activity was performed based on the study by de Menezes et al. (2021). Under the laboratory conditions of this study, the RIC_50_ value for the ascorbic acid solution was determined to be 0.276 mol/mol. The tested sample was prepared in five different concentrations using serial (binary) dilution. A total of 2 mL of the diluted sample and 4 mL of a freshly prepared methanolic DPPH solution (0.004 %) were added to a test tube. The reaction was allowed to proceed for 30 min at room temperature in a dark room. The absorbance of the solution at 515 nm was determined spectrophotometrically. From the resulting dependence, the amount of sample required to inhibit 50 % of DPPH (AIC_50_) was calculated. This value was then divided by the molar concentration of DPPH in the reaction. The final result was expressed as RIC_50_ in units of g/mol for ethanol extracts and mL/mol for essential oils. All determinations were performed in duplicate.

### 2.5 Dry fermented salami preparation

Dry fermented salami (lovecký salám) was prepared according to the recipe described in the book Czech Meat Products (Šedivý, 2019). Beef meat was minced using a cutter with 4.5 mm holes together with spices, a starter culture (BITEC LS-25-2 containing *Staphylococcus carnosus* and *Lactobacillus sakei*), and a nitrite curing mix. A plant extract was then added to the mixture, followed by the addition of pork meat. The complete mixture was minced again.

A total of nine batches were prepared: one control batch without any plant additives, and eight experimental batches containing either ethanol extracts or essential oils of sage and ginger. For each plant material, two types of extracts were used: ethanol extracts (ginger extracted in 80 % ethanol, sage in 60 % ethanol) and essential oils. Each extract type was applied in two concentrations: 0.05 % and 2.00 % for ethanol extracts, and 0.005 % and 0.010 % for essential oils. Each batch was produced in sufficient quantity to allow for all planned analyses throughout the five-week storage test.

The prepared mixture was stored at 2 °C until the following day. It was then stuffed into cellulose casings, with each salami weighing approximately 200 g. This was followed by shaping and an initial ripening phase at 4 °C for two days. After this phase, the salamis were treated with liquid smoke (spray-applied to the surface of the salami).

The salamis were then hung in a ripening chamber set at 14 °C, with a relative humidity maintained between 80 % and 90 %. The ripening process lasted for 14 days.

Following the ripening phase, a storage experiment was conducted. The salamis were stored at 18 °C and a relative humidity of 60 %. Over a five-week period (excluding the fourth week), samples were analyzed weekly. The assessments included lipid oxidation (TBARS), water activity, and color measurement.

### 2.6 TBARS assay for lipid oxidation in dry fermented salami

The TBARS (thiobarbituric acid reactive substances) determination was performed using the procedure published by Tarladgis et al. (1960). A total of 10 g (± 0.001 g) of a thoroughly homogenized sample from each salami was weighed into a distillation tube. Subsequently, 97.5 mL of distilled water and 2.5 mL of hydrochloric solution (water:HCl, 2:1, v/v) were added. The distillation tube was placed into a BÜCHI distillation unit, and a program was initiated with 30 % steam for 10 min. After the completion of distillation, approximately 50 mL of distillate was obtained. Between each sample, the instrument was flushed under the following conditions: 5 min, 6 % steam. The distillate was weighed, collected in a ground-glass Erlenmeyer flask, and stored sealed at 5 °C until the following day. The next day, 5 mL of the distillate and 5 mL of a 2-thiobarbituric acid solution (0.02 M, 90 % acetic acid) were transferred into a 400 mL boiling tube. The mixture was heated for 35 min in a water bath under a fume hood and subsequently cooled for 10 min. Absorbance at 538 nm was determined against the prepared blank using a UV–Vis spectrophotometer. The thiobarbituric acid number (*c*_*MDA*_), expressed as mg of malondialdehyde per kg of sample, was calculated according to Eq. 1:

(1) $$c_{\mathrm{MDA}}=7,8∙A∙\frac{10∙m_{D}}{50∙m_{S}\;},$$

where *A* is the measured absorbance, *m*_*D*_ is the weight of the distillate after distillation and *m*_*S*_ is the weight of the sample. All determinations were performed in duplicate.

### 2.7 Water activity measurement in dry fermented salami

Water activity was measured using the AQUALAB 4TEV device. The measurement was performed on a freshly cut whole slice of salami, as described in the study by Beňo et al. (2023). Each salami sample with the addition of a plant extract at different concentrations, as well as the control sample, was analyzed three times on each measurement day. All determinations were performed in triplicate.

### 2.8 Color measurement in dry fermented salami

The measurement was performed using a CM-5 spectrophotometer in SCE mode (specular component excluded) with an 8 mm measurement aperture. Prior to measurements, the device was calibrated with a white calibration standard and a black trap, following the manufacturer's instructions. Measurements were taken under standard illuminant D65. Two slices were cut from each salami sample, which was then wrapped in a transparent polyethylene film and measured at 10 different points. The color was recorded in L*a*b* coordinates using the SpectraMagic™ NX software. The total color difference (ΔE*_ab_) was calculated according to ISO/CIE 11664–4:2019 (International Commission on Illumination (CIE), and International Organization for Standardization (ISO), 2019) for each week of storage by comparing the control sample with each treatment.

### 2.9 Statistical analysis

The antioxidant properties of the plant extracts were evaluated using one-way ANOVA with repeated measures, followed by Tukey's post-hoc test (*p* < 0.05). The determination of total phenolic content was based on four measurements per sample, while the inhibition of DPPH was assessed using two sets of dilutions. The results of the storage experiment for the model meat product were analyzed using two-way ANOVA with repeated measures, followed by a post-hoc test with Bonferroni correction based on estimated marginal means (emmeans package in R) (*p* < 0.05). The determination of TBARS was based on two measurements per sample, water activity on three measurements per sample, while color analysis was performed using twenty measurements per sample. Results from all analyses are expressed as the mean of replicates ± standard deviation.

## 3 Result and discussion

### 3.1 Total phenolic content of plant extracts

**Fig. 1:**
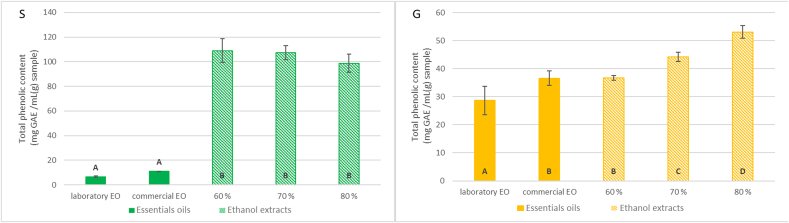
Title: Total phenolic content of sage (S) and ginger (G) extracts. Description: No statistically significant difference was observed between columns with the same index (*p* > 0.05).

The results of the total phenolic content determination are shown in Fig. 1. The highest total phenolic content was observed in ethanol extracts of sage, with no statistically significant difference between the ethanol concentrations Therefore, the 60 % ethanol extract was selected for further testing due to economic considerations. It contained 109 ± 10 mg GAE/1 g DW. For ginger, the highest phenolic content was obtained with the 80 % ethanol extract, which was statistically distinct from the other extracts, reaching 53.0 ± 2.3 mg GAE/1 g DW. These two extracts were subsequently prepared in larger quantities and utilized in an experiment involving the incorporation of plant extracts into a model meat product.

The reported total phenolic content in sage (*Salvia officinalis* L.) extracts varies considerably across different studies: 5.80 ± 1.00 mg GAE/g DW (Roby et al., 2013), 31.12 mg GAE/g DW (Safarpour et al., 2023), 38.12 mg GAE/g DW (Dent et al., 2024), 98.84 ± 38.05 mg GAE/g DW (Sonja Duletic et al., 2016) and 96.1 ± 2.6 mg GAE/g DW (Jasicka-Misiak et al., 2018). In another sage species (*Salvia sclarea*), the total phenolic content was reported to be 134.4 ± 9.7 mg GAE/g DW (Jasicka-Misiak et al., 2018). Numerous factors influence the chemical composition of plant extracts. These including geographical origin, ecological and climatic conditions, genetic variation of the plant, as well as the extraction conditions themselves (Jasicka-Misiak et al., 2018; Olszewska et al., 2020). The total phenolic content obtained in this study was slightly higher compared to the values reported in the aforementioned studies.

Various studies investigating the antioxidant activity of dried ginger have reported total phenolic content values ranging from 9.019 to 11.97 mg GAE/g dry weight: 10.099 ± 0.002 mg GAE/g DW (Ezez & Tefera, 2021), 9.019 ± 0.02 mg GAE/g DW (Ghafoor et al., 2020), and 11.97 ± 0.46 mg GAE/g DW (Mustafa & Chin, 2023). In this study, phenolic content was measured in the prepared extract, not in raw ginger. Given that the extraction yield in this experiment was approximately 20 %, it is reasonable to expect that the phenolic content in the extract would be roughly five times higher than the values reported in previous studies referring to whole ginger.

### 3.2 Antioxidant activity of plant extracts (DPPH assay)

**Fig. 2:**
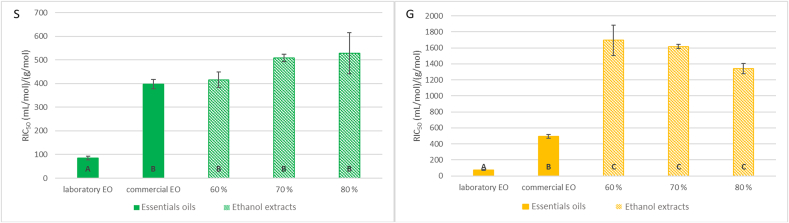
Title: DPPH radical-scavenging activity of sage (S) and ginger (G) extracts. Description: No statistically significant difference was observed between columns with the same index (*p* > 0.05).

The results of the determination of DPPH radical-scavenging activity are presented in Fig. 2. The highest antioxidant activity, indicated by the lowest relative half-maximal inhibitory concentration (RIC_50_), was observed in the laboratory-prepared essential extracts for both sage and ginger (sage: 85 ± 9 mL/mol, ginger: 73 ± 8 mL/mol). Both extracts exhibited statistically significant differences compared to other extract types (*p* < 0.05). They were subsequently prepared in larger quantities and utilized in an experiment involving the incorporation of plant extracts into a model meat product.

The reported IC₅₀ values for DPPH radical scavenging activity vary significantly in the literature. For sage essential oils, values range from 1.78 μL/mL (Bozin et al., 2007), to 8.31 ± 0.55 mg/L (El Euch et al., 2019), and up to 970 ± 5.5 mg/mL (El Jery et al., 2020). For ginger extracts, values include 0.64 μg/mL (Stoilova et al., 2007), 675 μg/mL (Höferl et al., 2015), and 1071.014 μg/mL (Sutrisno, & Moh. Ajirul Abiq., 2023). In a study by de Menezes et al. (2021), several inconsistencies were identified in the interpretation of DPPH assay results, particularly regarding the incorrect expression of IC₅₀ values. A common mistake is the failure to account for the concentration of DPPH^•^ when calculating antioxidant capacity, whereas the more appropriate approach would be to express the results as a molar ratio of antioxidant to DPPH^•^. As a result, comparing data across different studies is highly challenging (de Menezes et al., 2021). To the best of our knowledge, no studies have reported IC₅₀ values for sage and ginger extracts in a standardized and comparable format, such as RIC₅₀.

### 3.3 Lipid oxidation levels in dry fermented salami (TBARS)

**Fig. 3:**
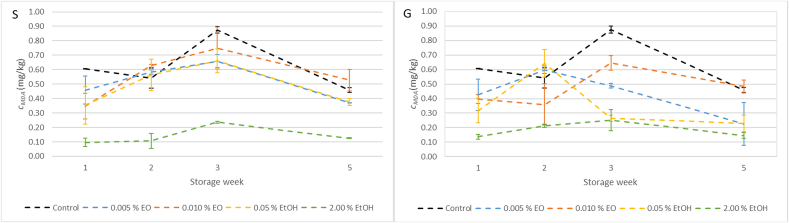
Title: Malondialdehyde levels in fermented salami with sage (S) and ginger (G) extract during a five-week storage test. Description: Data for the fourth week were not collected.

The results of malondialdehyde concentration in fermented meat product are presented in Fig. 3. A statistically significant effect of the applied additive, the time factor, and their interaction was observed for both extracts (*p* < 0.05). For sage extracts, a statistically significant difference was found only for the meat product with a 2 % addition of ethanolic extract (*p* < 0.05). This sample differed from all other samples, including the control. For ginger extracts, a statistically significant difference was observed between all samples (*p* < 0.05) except between the samples with 0.005 % and 0.010 % essential oil.

Several studies have investigated the antioxidative effects of sage and ginger extracts or essential oils in meat products; however, results remain difficult to compare due to inconsistencies in methodology and data reporting. For instance, Šojić et al. (2021) observed a statistically significant reduction in lipid oxidation in salamis supplemented with 0.01, 0.05, and 0.1 μL/g of sage essential oil (approximately 0.001 %, 0.005 %, and 0.010 %, respectively). In contrast, this study did not observe any statistically significant effect at either 0.005 % or 0.010 % levels of supplementation. Both the model meat products in the study Šojić et al. (2021) and in our investigation were fermented dry salamis. However, a key difference may lie in the fat content of the formulations—in Šojić et al. (2021), the fat content ranged between 31.1–33.4 % and 35.5–37.4 %. In contrast, the final fat content in our study was approximately 48 %, mainly due to more extensive moisture loss during ripening. This difference in composition may have contributed to the varying effectiveness of the added sage essential oil.

Similarly, in an earlier study, Šojić et al. (2018) reported a statistically significant inhibition of lipid oxidation in fresh salamis after 6 and 8 days of refrigerated storage with sage essential oil added at 0.05, 0.075, and 0.1 μL/g. The authors also reported an IC₅₀ value of 0.4823 mg/mL for the essential oil. However, their DPPH assay protocol described in their study lacks crucial methodological details, particularly regarding the concentration of DPPH^•^ used in the reaction system. This omission complicates any direct comparison of IC₅₀ values between studies. In this study, the AIC₅₀ value of the essential oil was calculated as 5.76 mL/L. However, due to the lack of standardized assay conditions—particularly concerning the DPPH^•^ to antioxidant ratio—and the insufficient methodological detail in the study by Šojić et al. (2018), a direct and meaningful comparison of IC₅₀ values is not feasible.

In a study by Cegiełka et al. (2022), both 70 % ethanol extracts and essential oils of sage were incorporated into chicken meatballs stored under refrigeration. After one week, only the sample supplemented with 2 % ethanol extract showed a statistically significant reduction in malondialdehyde levels compared to the control—findings consistent with our present study.

Olatidoye et al. (2015) tested ethanol extracts of ginger at concentrations of 0.2–0.8 % (based on dry matter) in minced beef. All tested concentrations were effective in controlling lipid oxidation throughout the 8-day storage period. In another study, Abdel-Naeem and Mohamed (2016) demonstrated that camel meat burgers with 7 % aqueous ginger extract exhibited significantly lower TBARS values compared to the control group during three months of frozen storage.

The observed antioxidant effects can be attributed to the presence of known bioactive compounds in the applied extracts. In sage, carnosic acid and carnosol are considered the primary phenolic diterpenes responsible for radical scavenging and lipid stabilization, along with monoterpenes such as α-pinene and eucalyptol that also exhibit antimicrobial and antioxidant properties (Yazgan, 2020; D. Zhang et al., 2022).

These compounds act through distinct antioxidative mechanisms, as described in previous research. Carnosic acid primarily functions as a chemical quencher of reactive oxygen species (ROS) such as singlet oxygen and hydroxyl radicals, and is subsequently oxidized to carnosol. In contrast, carnosol exhibits antioxidant activity through a different mechanism that does not rely on ROS scavenging. It has been shown to inhibit lipid peroxidation directly, even under oxidative conditions that do not involve ROS generation (Loussouarn et al., 2017).

Ginger is rich in phenolic compounds such as 6-gingerol, 6-shogaol, and zingerone, which are known for their radical scavenging, antimicrobial, and color-preserving activities in various food systems (Beristain-Bauza et al., 2019; D. Zhang et al., 2022). Specifically, 6-gingerol exhibits antioxidant effects primarily through electron donation and radical scavenging mechanisms (Sharma et al., 2022). Likewise, 6-shogaol has demonstrated strong DPPH• radical scavenging activity, indicating its high antioxidant potency (Bischoff-Kont & Fürst, 2021). The observed differences in antioxidant efficacy between ethanol extracts and essential oils may stem from variations in compound polarity, volatility, and their distribution and reactivity within the meat matrix.

In summary, plant extracts, especially from sage and ginger, have potential to reduce lipid oxidation in meat. However, few studies provide reproducible and well-documented methods, making comparisons across studies difficult. There is a clear need for standardization in assay protocols and reporting units to improve the interpretability and applicability of future research in this field.

### 3.4 Changes in water activity of dry fermented salami during storage

**Fig. 4:**
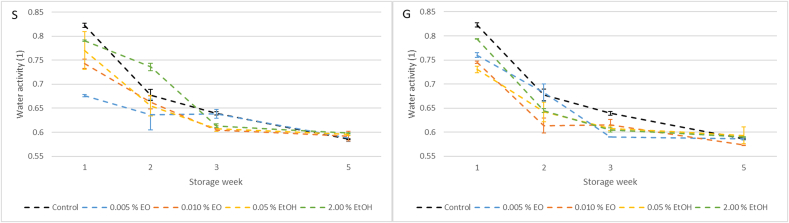
Title: Water activity in fermented salami with sage (S) and ginger (G) extract during a five-week storage test. Description: Data for the fourth week were not collected.

The results of water activity measurements in the dry fermented salami are presented in Fig. 4. A statistically significant effect of the applied additive, the time factor, and their interaction was observed for both extracts (*p* < 0.05). Among sage treatments, only the 0.005 % essential oil concentration differed significantly from the control (p < 0.05). In contrast, all tested concentrations of ginger extracts showed a statistically significant difference compared to the control sample (*p* < 0.05). During storage weeks two and three, water activity decreased noticeably, averaging a drop of approximately 0.13. This decline is most likely attributable to suboptimal storage conditions—specifically, a temperature of 18 °C and a relative humidity of 60 %, which may have promoted excessive moisture loss.

When comparing these results to other studies, notable differences in water activity values emerge, likely due to divergent storage parameters. For instance, Dasiewicz et al. (2024) monitored water activity in salami-type meat products during storage for up to 63 days and reported stable values around 0.85. Their samples were stored at 2–4 °C and 70–80 % relative humidity, with a fat content of 42 %. Similarly, Stangierski et al. (2023) observed water activity levels between 0.89 and 0.87 over a 45-day storage period at 10 °C. Their product, a smoked salami with 32 % fat content, was packed in plastic bags in a modified atmosphere. Rubio et al. (2007) also reported water activity values around 0.86 in dry fermented salami stored for 150 days at 6 °C, likewise packaged in plastic bags. Andrés et al. (2006) reported no significant effect of fat content on water activity in meat products.

In contrast to these controlled conditions, the elevated storage temperature and lower humidity used in the present study likely contributed to more pronounced moisture loss, resulting in water activity values ranging from 0.60 to 0.80. These values fall outside the typically reported range of 0.85–0.89 considered desirable for DFS, thus suggesting that the storage conditions employed here were not optimal. This highlights the need for stricter control of production parameters in the laboratory environment to ensure more consistent product quality. The unexpectedly low water activity values may have further influenced the antioxidant efficacy of the plant extracts. According to previous findings (Nelson & Labuza, 1992), lipid oxidation in food systems may accelerate under certain intermediate water activity levels, particularly within the 0.60–0.80 range. Therefore, the reduced antioxidant effect observed in some samples of this study may partially result from unfavorable physicochemical conditions during storage, rather than solely from the nature or concentration of the extracts themselves.

### 3.5 Color development in dry fermented salami

**Fig. 5:**
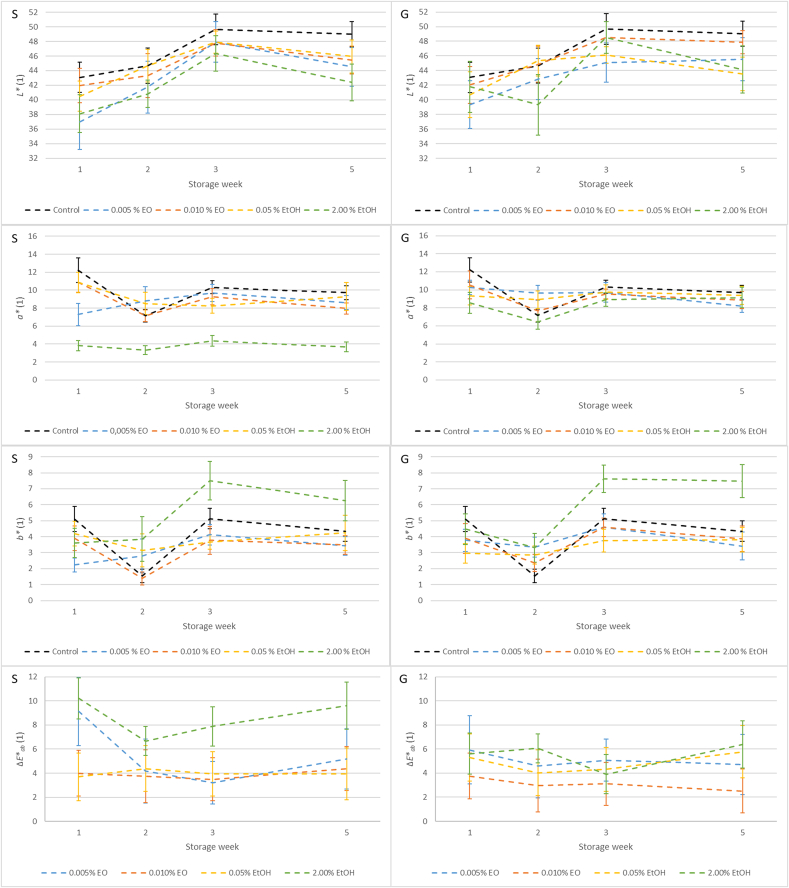
Title: Color parameters of lightness (L*), red chromaticity (a*), yellow chromaticity (b*), and the total color difference (ΔE*_ab_) of fermented salami with sage (S) and (G) ginger extracts during five-week storage. Description: Data for the fourth week were not collected.

The results of lightness (L*), red chromaticity (a*), yellow chromaticity (b*), and the total color difference (ΔE*_ab_) of fermented meat product are presented in Fig. 5. A statistically significant effect of the applied additive, storage time, and their interaction was observed for lightness in both extract types (*p* < 0.05). In salami treated with sage extracts, all samples significantly differed from the control. Similarly, in the ginger group, significant differences were found between the control and all other treatments (*p* < 0.05), except for the sample containing 0.010 % essential oil.

Red chromaticity was also significantly affected by the additive, time, and their interaction (*p* < 0.05) for both extract types. In salami with sage extracts, all treatments showed statistically significant differences compared to the control. For ginger-treated salami, all treatments differed significantly from the control (*p* < 0.05), with the exception of the sample containing 0.005 % essential oil.

A similar trend was observed for yellow chromaticity, where the effect of the additive, time, and their interaction was statistically significant in both extract groups (*p* < 0.05). In the sage group, all treatments differed significantly from the control, except the sample with 0.05 % ethanolic extract. In the ginger group, significant differences were recorded for all treatments compared to the control (*p* < 0.05), except for the sample with 0.005 % essential oil.

In all samples and at all time points, the total color difference exceeded one unit, which is generally considered perceptible to the human eye (Altmann et al., 2022).

The addition of sage extracts led to a reduction in lightness (L*), consistent with the findings of Šojić et al. (2021). This effect is most likely caused by interactions between myoglobin and bioactive compounds present in sage essential oil, such as terpenoids and phenolics (Šojić et al., 2021), or by the presence of pigmented plant constituents like chlorophylls (L. Zhang et al., 2013). The sample treated with 2.00 % ethanolic sage extract showed the most pronounced color difference, including a shift towards the green spectrum (a*). This level of discoloration may already be considered undesirable from a sensory perspective.

## 4 Conclusion

This study demonstrated the potential of ethanol extracts and essential oil from sage (*Salvia officinalis* L.) and ginger (*Zingiber officinale*) as effective natural antioxidants in dry fermented salami. The highest total phenolic content among the tested samples was observed in 60 % ethanol extract of sage and 80 % ethanol extract of ginger, both of which were selected for further application in meat product. Antioxidant capacity, evaluated by DPPH radical-scavenging activity, was greatest in the essential oil extracts. Incorporation of these extracts into a dry fermented salami confirmed their antioxidative efficacy, particularly at higher concentrations. A significant reduction in malondialdehyde formation was observed with 2 % sage ethanol extract and all tested concentrations of ginger extracts, confirming their antioxidative efficacy in a real food system. However, sage essential oil concentrations of 0.005 % and 0.010 % did not produce statistically significant effects. Overall, these findings support the application of sage and ginger extracts as promising clean-label alternatives to synthetic antioxidants in fermented meat products. Further research should address sensory attributes and consumer acceptance to evaluate commercial viability.

## CRediT authorship contribution statement

**Filip Hruška:** Writing – original draft, Visualization, Project administration, Methodology, Investigation, Funding acquisition, Formal analysis, Conceptualization. **Filip Beňo:** Writing – review & editing, Methodology, Investigation. **Gabriela Krátká:** Writing – review & editing, Investigation. **Rudolf Ševčík:** Writing – review & editing, Supervision, Methodology, Funding acquisition.

## Declaration of generative AI and AI-assisted technologies in the writing process

During the preparation of this work the authors used ChatGPT (OpenAI) in order to improve the readability and clarity of the English language in the manuscript. In addition, AI tools were utilized to assist in the creation of the graphical abstract. After using this tool, the authors reviewed and edited the content as needed and takes full responsibility for the content of the published article.

## Funding sources

This work was supported by the grants of Specific university research, grant No A2_FPBT_2024_027 and grant No A1_FPBT_2025_002, funded by the [University of Chemistry and Technology, Prague].

## Declaration of competing interest

The authors declare that they have no known competing financial interests or personal relationships that could have appeared to influence the work reported in this paper.

## Data Availability

Data will be made available on request.
